# Endoscopic Biopsies and Histopathological Findings in Diagnosing Chronic Gastrointestinal Disorders in Dogs and Cats

**DOI:** 10.1155/2020/8827538

**Published:** 2020-10-09

**Authors:** Andrzej Rychlik, Ewa Kaczmar

**Affiliations:** Department of Clinical Diagnostics, Faculty of Veterinary Medicine, University of Warmia and Mazury, Oczapowskiego 14, Olsztyn 10-719, Poland

## Abstract

Nowadays, endoscopic examination is a diagnostic tool gaining popularity in the management of gastrointestinal disorders in dogs and cats. Direct accessibility of the lumen of gastrointestinal tract combined with the mucosal biopsy provides a great diagnostic potential. Using endoscopy and endoscopically guided biopsy, one can conduct both macro- and microscopic assessment of lesions and perform many specialist adjunct examinations. Histopathological examination of mucosal biopsy specimens collected from the stomach and intestines allows us to distinguish between types of inflammations and to diagnose ulcerative, polypoid, and cancerous lesions.

## 1. Introduction

Gastroduodenoscopy or colonoscopy in combination with endoscopically guided biopsy is regarded as one of the most useful techniques used in diagnosing chronic inflammatory diseases of the gastrointestinal tract in dogs and cats [1, 2]. An endoscopic examination enables a macroscopic assessment of the accessible parts of the gastrointestinal tract and collecting the biopsy specimens from the mucous membrane of the oesophagus, stomach, and intestines, as well as from polyps and tumours. According to the results of studies conducted in our endoscopic laboratory and literature data, the most frequent endoscopic lesions revealed in such examinations include oedema of the mucous membrane, mucosal thickening, hyperaemia, extravasations, and erosion of gastrointestinal tract sections [3–6]. During an endoscopic examination, samples of mucosa for histopathological analysis are collected with suitable instruments introduced through the working channel of the endoscope. Collected biopsy specimens can be used to determine the degree of inflammation, differentiate inflammatory and neoplastic lesions (benign and malignant), and assess any applied treatment. Lesions of the gastrointestinal mucosa are frequently patchy, and not all biopsies may be adequate diagnostically, which is why it is recommended to collect six to eight specimens from each gastrointestinal tract part [7]. A histopathological examination involves assessment of the epithelium, lamina propria, crypts, and intestinal villi, together with an analysis of the intensity of inflammatory cell infiltration, conducted to determine the type and intensity of the inflammatory process [2, 8]. The collected samples are routinely examined histopathologically, but they can also be used in such procedures as electron microscopic assessment and immunohistochemical or cytological examinations [9, 10]. Surgical methods of collecting biopsy specimens for examinations require tedious and costly procedures that do not always result in a certain diagnosis. Laparotomy may enable collection of a whole fragment of the gastric or intestinal wall but compared to endoscopy, it is a highly invasive method, as the patient is subjected to a full procedure of abdominal surgery. However, when neoplastic lesions are suspected, laparoscopy or laparotomy with collecting a specimen of the whole gastric or intestinal wall is recommended [11, 12]. An endoscopic examination with collecting specimens for a histopathological examination is the basis for diagnosing chronic enteropathies, without their differentiation. A diagnosis of the type of a chronic enteropathy in a patient is based on the response to treatment or on additional laboratory tests [2, 13].

## 2. Histopathological Examination of Biopsy Specimens in Enteropathies

The endoscopic biopsy of the intestine is regarded as the gold standard in the diagnosis of IBD in dogs and cats [6, 7, 10, 14–18]. The Gastrointestinal Standardization Group of WSAVA proposed a standard of histopathological assessment of biopsy specimens in 2008 [7]. According to its recommendations, changes in the morphology of various elements of mucosa should be analysed during the histopathological evaluation and the number of cells in the *lamina propria* region should be determined. Analysis of biopsy specimen of submucosa is of limited importance because of low accessibility, while performing endoscopic biopsy [19]. The histopathological assessment takes into account such mucosal structures as the epithelium (size of enterocytes, structure continuity, number and size of goblet cells, and cellular infiltration), lamina propria (cellular infiltration, fibrosis, and dilation of lymphatic vessels), crypts (crypt depth, hyperplasia, proliferation and differentiation of goblet cells, and presence of inflammatory cells), and intestinal villi (villi structure—length, width, and shape). An analysis of the structural integrity involves an assessment of whether excessive exfoliation, defects, and corrosion are present, i.e., fresh mucosal defects without the surrounding cellular reaction. It is also important to determine the degree of epithelium damage and how many damaged villi there are [7, 10, 17, 19–21]. The criteria of the mucosal histopathological assessments are shown in Table 1. Notable features of the histopathological image include the bacterial count on the epithelium surface. IBD is often associated with changes in the intestinal microbiome. The presence of *Escherichia coli* and *Campylobacter* has been emphasised recently, as they can be an important factor that disrupts the proper immunological status of the intestines [22, 23]. However, these cannot be definitively identified by routine histology.

Despite its indisputable diagnostic value, the histopathological examination of biopsy specimens has some limitations. Histopathological lesions of the gastrointestinal mucosa are poorly correlated with an assessment of the clinical response to the applied therapy, and their application in diagnosis is limited [20, 24–26]. Similar to clinical indices, this examination does not distinguish food-responsive enteropathies, bacterial hypertrophy, or parasitic enteropathy from IBD [3, 4, 27]. All these chronic enteropathies usually feature infiltration of mononuclear cells in the *lamina propria* and lesions shown in Table 1. However, the definitive diagnosis is based on the effects of treatment (including elimination diet).

## 3. Histopathologic Findings

A histopathological examination aims to distinguish between normal and pathological tissue, determine the type and intensity of lesions in the mucosa, facilitate a correct diagnosis, and start the appropriate treatment. Although endoscopic biopsy and a histological examination of biopsy specimens are effective in diagnosing only those lesions, which affect the mucosa, the high diagnostic utility of the technique must be also clearly stressed.

## 4. Inflammatory Lesions

**Figure 1:**
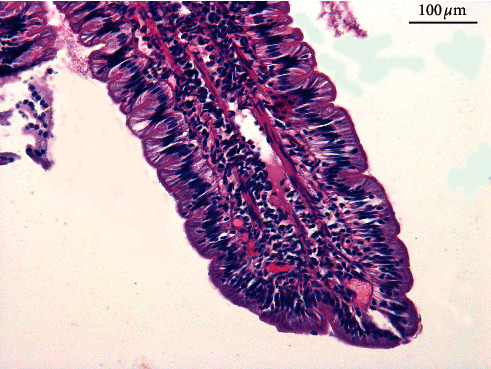
The microscopic view of infiltration of inflammatory cells observed in LPE.

**Figure 2:**
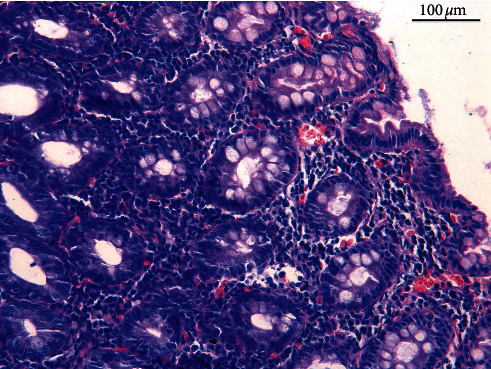
The microscopic view of infiltration of inflammatory cells observed in LPC.

Inflammatory lesions are the most frequent findings in biopsy specimens collected from the canine and feline gastrointestinal tract [14, 28–30]. Unfortunately, the interpretation of these lesions is often not easy. The work of numerous independent authors has resulted in developing a system of classification and intensity of inflammatory lesions. The majority of researchers have agreed that the determination of the type of lesions should be based on the identification of the dominant cell population in the inflammatory infiltration in the *lamina propria* (e.g., lymphocytes, plasma cells, eosinophils, neutrophils, and macrophages) and the process intensity should be described in a 4-point scale (N—no lesions, +—mild lesions, ++—moderate lesions, and +++—severe lesions) [3, 4, 7, 15, 26, 31]. The most frequent in chronic enteropathies is lymphocytic-plasmacytic enteritis (LPE) (Figure 1) or lymphocytic-plasmacytic colitis (LPC) (Figure 2). The eosinophilic form, more frequently encountered in the food-responsive enteropathy (FRE), is typically a more severe form of inflammation, accompanied by erosions and ulcers [32]. There are also other forms of cellular infiltration in the lamina propria region, e.g., granulomatous colitis (GC), also referred to as histiocytic ulcerative colitis (HUC) [33–35]. Until recently, it was believed that the inflammatory bowel disease (IBD) was the most frequent chronic enteropathy, but a study conducted by Allenspach on 203 dogs with chronic enteropathy demonstrated that the food-responsive enteropathy (FRE) was diagnosed more frequently [36]. However, IBD diagnosis is based not only on the histopathological confirmation of the mucous membrane inflammation in the collected biopsy specimens. The other factors such as (1) persistent or recurrent signs from the gastrointestinal tract (diarrhoea and vomiting) for more than 3 weeks, (2) no evidence of other causes of the inflammation (exclusion of systemic diseases by laboratory and imaging examinations), (3) poor or no response to a change in the diet (FRE), antiparasitic medicines, and antibiotics, and (4) improvement of the clinical condition following administration of anti-inflammatory or immunosuppressive drugs must also be present [17]. This last factor is a consequence of the fact that IBD is probably caused by altered interactions between intestinal microorganisms and the mucosal immune system. Such susceptible individuals develop a strong immune response due to modification of the intestinal microbiome [37–39]. However, it must be noted that the immunosuppressive treatment is ineffective in some animals with suspected IBD and in those with excluded enteropathies (non-steroid responsive enteropathy, non-SRE patient). It is a consequence of the fact that IBD is an idiopathic disease, in which the etiopathogenesis is not fully understood.

## 5. Ulcerative Lesions

Ulcerative lesions in the canine and feline gastrointestinal tract are typically located in the gastric and intestinal mucosa. They can be a consequence of acute gastrointestinal inflammation of various aetiologies, and they are usually caused by neoplastic processes or long-term administration of glucocorticosteroids or NSAIDs [40, 41]. Ulcers caused by NSAIDs resemble conical craters, which may be accompanied by erosions. Ulcers located in the stomach or proximal duodenum are classified as peptic ulcers, sharply demarcated from the surrounding area, covered with considerable cellular debris and purulent discharge with an admixture of blood. Therefore, it is important to remove the accumulated mass with a strong jet of water before biopsy. Ulcers located in the *incisura angularis* of the stomach are often cancerous, usually an adenocarcinoma. In our clinical practice, we have observed ulcerative lesions located near the cardia in dogs. The occurrence of ulcerative parasitic gastritis, which is caused by the nematode *Aonchotheca putorii*, has been documented in cats [42]. Biopsy specimens collected from the central and surface parts of ulcers are usually useless and mainly contain necrotic and inflammatory debris regardless of the primary cause of the condition. The tissue should be collected several times from the same place (deep biopsy), preferably on the ulcer edges, especially when the neoplastic background of the lesions is suspected. Furthermore, the tissue surrounding the ulcers, even if it looks normal, should be collected to exclude histopathological lesions, despite a normal endoscopic appearance [43].

## 6. Neoplastic Lesions

**Figure 3:**
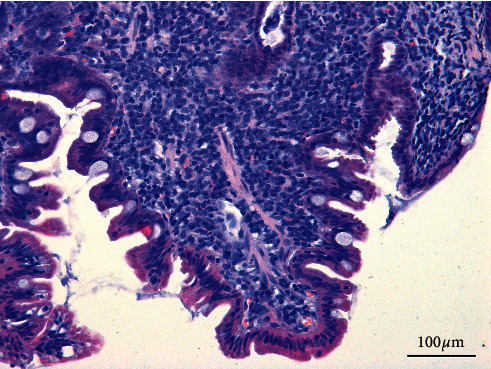
The microscopic view of infiltration of inflammatory cells observed in alimentary lymphoma in cat.

Alimentary lymphoma is one of the most frequently diagnosed gastrointestinal cancers in small animals, especially in cats. Alimentary lymphoma can occur in the upper and in the lower parts of the gastrointestinal tract as cancerous infiltrations or tumours. *Helicobacter* infections are suspected to play a significant role in lymphoma development in cats. One study found that *Helicobacter heilmannii* was found in gastric biopsy specimens in 16 out of 24 cats with lymphoma [44]. However, *Helicobacter* can be found in up to 100% of cats in a variety of studies [44]. Chronic lymphocytic-plasmacytic inflammations of gastrointestinal mucosa are known to be precursors of this condition in humans and animals [45]. Therefore, the problem of distinguishing this IBD form in cats and dogs from lymphoma remains. It is difficult to state without a doubt based on a histopathological picture, whether a lesion is an intensive chronic lymphocytic-plasmacytic inflammation or a lymphoid neoplasm, especially when the size, number, or quality of biopsy specimen is not satisfactory. Therefore, it is recommended that histopathological assessment of biopsy specimen should be conducted by at least two veterinary histopathologists or by an experienced histopathologist who has been collaborating with an entity providing biopsy specimens for analysis for a long time. This will ensure higher objectivity and diagnostic value of cooperation between the gastroenterologist and the histopathologist. If it is difficult to distinguish between neoplastic and inflammatory lesions, additional (e.g., immunohistochemical) tests of biopsy samples should be performed [46, 47]. It may be difficult to assess histopathological specimens properly because considerable infiltration of inflammatory cells can often be observed in alimentary lymphoma (Figure 3). On the other hand, the lymphoma which develops in the gastric wall is often accompanied by ulcerative lesions and secondary inflammation, and neoplastic cells are located deep in the submucosal layer [48, 49]. Endoscopic biopsy specimens rarely contain deep submucosal tissue, which is why diagnosing the lymphoma or other neoplasms with this technique may have a considerable error [50]. The experience of the endoscopic laboratory of the Faculty of Veterinary Medicine, UWM in Olsztyn, shows that neoplastic lesions in the stomach usually start spreading from the serous membrane. Therefore, it is very important to perform indirect imaging examinations, such as ultrasound or CT scan, which may locate the developing neoplastic process in the gastric wall. If neoplastic lesions are suspected and imaging examinations cannot be performed or if they give inconclusive results, laparotomy or laparoscopy with collecting a specimen of the whole intestinal wall is recommended. It can help to distinguish cancer in an endoscopic examination in which—according to some studies—the lymphoma is not accompanied by the oedema and inflammation of the mucous membrane, which are typical of IBD [51]. These lesions are visible in an endoscopically guided biopsy, and they should be included in a description of the material for histopathological examinations (Table 1). Moreover, compared to IBD, neoplastic lesions are usually associated with enlarged lymph nodes in the abdominal cavity. A blood smear assessment can provide valuable results. Anaemia and morphological changes in blood cells have been observed more often in lymphomas compared to IBD patients [52].

**Figure 4:**
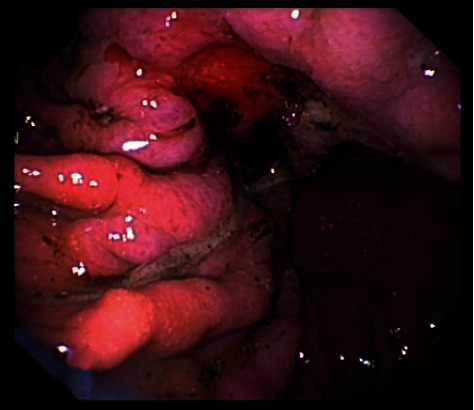
The endoscopic view of adenocarcinoma in canine stomach.

Apart from lymphoma, frequent gastrointestinal tumours in dogs and cats, diagnosed based on a histopathological examination of biopsy specimens, include carcinomas, adenocarcinomas, gastrointestinal stromal tumours (GISTs), leiomyomas, and leiomyosarcomas [53–56]. Gastric cancers in dogs are rare, and they account for less than 1% of all tumours. The majority of gastric tumours are malignant, and half of them are adenocarcinomas (Figure 4). Malignant tumours are the most frequently diagnosed in intestines of small animals. The majority of them are adenocarcinomas in dogs and alimentary lymphomas in cats, usually in the form of intestinal infiltrations. Neoplastic masses are rare in the small intestines [55, 57]. Gastrointestinal cancers are usually diagnosed in older animals. The average age is 9 years in dogs and 11 in cats [55, 56]. Although gastrointestinal tumours are found rarely in dogs and cats, the large intestine is their most frequent location. Colorectal cancers account for 36–60% of all alimentary cancers in dogs and 10–15% in cats [58]. Squamous cell carcinomas (SCC), osteosarcomas (OSA), fibrosarcomas (FSA), and leiomyosarcomas are rarely found in the oesophagus in dogs [59, 60].

## 7. Polypoid Lesions

Endoscopic examinations of the alimentary tract of small animals may also reveal the presence of polyps, usually in dogs near the anorectal junction or several centimetres into the rectum. Asian cat breeds, such as Siamese or Himalayan cats, have been found to be more prone to develop polyps in the duodenum [53]. These are usually benign adenomas, but a histopathological examination of the biopsy specimen may also indicate early phases of malignancy [61]. It is typical of polypous lesions in the large intestine that there is no invasion into the muscular layers, which is the case in malignant tumours. Unfortunately, in such cases, it is not usually possible to make the diagnosis from a histopathological examination of biopsy specimen which does not include the intestinal muscular layers. Polyps can be single or multiple, and they are usually peduncular or sessile. Small polyps (<5 mm) can be removed with biopsy forceps or hot biopsy forceps with single or multiple biopsies [62, 63]. However, it has been found in some studies that such procedures may be an irritant in humans, provoking polyps to malignant transformation [64, 65]. Polypectomy of small polyps (2–7 mm) performed with small (11–13 mm) cold snares is the most frequent and the safest method [63, 66, 67].

## 8. Atrophic Lesions

Atrophic lesions in the mucosa usually occur in the autoimmune-induced metaplastic inflammation. This type of inflammation is associated with the presence of antibodies against gastric lining cells and against an internal factor. Damage in lining cells causes anacidity of gastric juice and B_12_ vitamin absorption disorders in humans. Upon entering the organ, the endoscopic view shows that gastric folds are poorly visible, whereas the blood vessel network is visible clearly. The endoscopic view shows a decreased number of lining cells. It is possible to assess the degree of atrophy of intestinal villi and the change of their shape in moderate and severe chronic inflammations of the small intestine with good quality and proper orientation of a section [7, 17].

## 9. Hyperplastic Lesions

**Figure 5:**
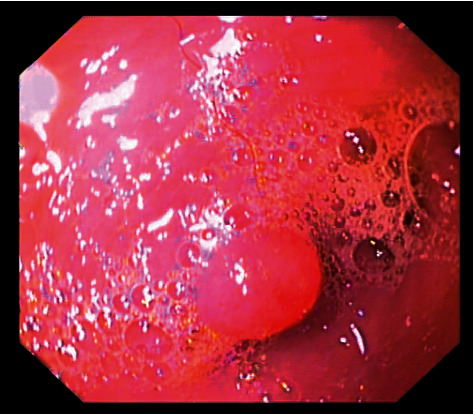
The endoscopic view of hyperplastic lesion in the pyloric canal of the dog.

Features of hyperplastic lesions of the mucosa are associated with many gastrointestinal tract conditions. The most frequent conditions include hypertrophic pyloric gastropathy in dogs [68], adenomatous polyposis in dogs and cats [53], and Zollinger–Ellison syndrome associated with excessive gastrin secretion by gastrinomas in the canine pancreas [68]. The endoscopic view of the stomach usually features mucosal thickening and only a slight reduction of the gastric wall folds following insufflation. Hyperplastic lesions can be focal, usually restricted to the pyloric antrum or the pyloric canal (Figure 5) or disseminated in different parts of the stomach or rarely, intestines. This type of inflammation in the pyloric part of the stomach is usually a result of duodenogastric reflux. The structure of the mucosa in the endoscopic view is granular and white-grey in colour as a result of blood vessel atrophy and connective tissue hypertrophy. Biopsy specimens for histopathological examination should be collected from the cardiac, body, or pyloric antrum region and the type of lesion should be described, e.g., polyp and thickened mucous membrane. Hyperplastic lesions are visible in the endoscopic assessment of the stomach and the intestines, but it should be noted that thickening of the gastric mucosa can also result from oedema caused, e.g., by vomiting.

## 10. Vascular and Lymphatic Lesions

Endoscopic examinations of the small intestines in dogs can reveal characteristic multifocal lesions on the mucosal surface. They look like multiple, light-coloured lumps (resembling an image of reflected light), often covered with mucous discharge. The histopathological evaluation of the mucosa reveals a considerable dilation of the lymphatic vessels of intestinal villi as empty spaces vacated by proteins. These lesions confirm the presence of lymphangiectasia. It is a congenital or acquired disease which involves disruption of the lymphatic system function in the small intestine, which can result in protein-losing enteropathy. Lymphangiectasia occurs in dogs of every age, and it is usually associated with inflammation [69, 70]. Large intestine blood vessel disorders called vascular ectasia or angiodysplasia are very rarely found in dogs [71, 72]. An endoscopic examination shows multiple multifocal erythema of vascular tissue, which cause rectal bleeding or melena. Biopsy specimens collected from altered parts show characteristic histopathological lesions. These are mainly considerably dilated blood vessels in the lamina propria of the mucous membrane and perivascular lymphocytic cell infiltrations [72].

## 11. Conclusions

Endoscopic biopsies of the gastrointestinal tract in dogs and cats are minimally invasive procedures, which do not require further hospitalisation or convalescence of the patients. Applying multiple-use tools makes these procedures cheaper and more common in routine veterinary medicine. This paper shows that the utility of endoscopy in diagnosing and differentiating the causes of gastrointestinal diseases disorders is very high. It describes only the most common pathological lesions. Endoscopically guided biopsy remains the main diagnostic technique in some gastrointestinal tract diseases, including chronic enteropathies.

## Figures and Tables

**Table 1:** Criteria for histopathological evaluation of the GI lining.

Structure	Classification of lesions
I—epithelium	
*Size of enterocytes*	No lesions (0) to significantly changed (3)
*Continuity of structure*	No damage (0) to significantly damage (3)
*Number and size of goblet cells/100 enterocytes*	Normal (0) to significantly changed, large quantity of mucus (3)
*Cellular infiltration*	None (0) to significant infiltration (3)
II— lamina propria	
*Cellular infiltration*	None (0) to significant infiltration (3)
*Fibrosis*	None (0) to significant fibrosis (3)
*Lymphangiectasia*	None (0) to significant lymphangiectasia (3)
III—crypts	
*Atrophy*	None (0) to significant atrophy (3)
*Hypertrophy*—*shape change*	None (0) to significant hypertrophy (3)
*Proliferation*— *hyperplasia*	None (0) to significant proliferation (3)
*Diversification of goblet cells*	None (0) to significant diversification (3)
*Presence of inflammatory cells*	None (0) to significant infiltration (3)
IV—villi	
*Structure (length, width, and shape)*	Normal (0) to significant change in structure (3)

## Data Availability

The data used to support the findings of this study are included within the article.

## Abbreviations

ACVIM: American College of Veterinary Internal Medicine
ARE: Antibiotic-responsive enteropathy
CIBDAI: Canine inflammatory bowel disease activity index
IBD: Inflammatory bowel disease
FRE: Food-responsive enteropathy
WSAVA: World Small Animal Veterinary Association.

## References

[1] Fefferman D. S., Farrell R. J. (2005). Endoscopy in inflammatory bowel disease: indications, surveillance, and use in clinical practice. *Clinical Gastroenterology and Hepatology*.

[2] Jergens A. E., Simpson K. W. (2012). Inflammatory bowel disease in veterinary medicine. *Frontiers in Bioscience*.

[3] Allenspach K., Wieland B., Gröne A., Gaschen F. (2007). Chronic enteropathies in dogs: evaluation of risk factors for negative outcome. *Journal of Veterinary Internal Medicine*.

[4] Garcia-Sancho M., Rodríguez-Franco F., Sainz A., Mancho C., Rodríguez A. (2007). Evaluation of clinical, macroscopic, and histopathologic response to treatment in nonhypoproteinemic dogs with lymphocytic-plasmacytic enteritis. *Journal of Veterinary Internal Medicine*.

[5] Jergens A. E., Crandell J. M., Evans R., Ackermann M., Miles K. G., Wang C. (2010). A clinical index for disease activity in cats with chronic enteropathy. *Journal of Veterinary Internal Medicine*.

[6] Rychlik A., Nieradka R., Kander M., Depta A., Nowicki M., Sarti K. (2007). Usefulness of endoscopic examination for the diagnosis of inflammatory bowel disease in the dog. *Polish Journal of Veterinary Sciences*.

[7] Day M. J., Blizer T., Mansell J. (2008). Histopathological standards for the diagnosis of gastrointestinal inflammation in endoscopic biopsy samples from the dog and cat: a report from the world small animal veterinary association gastrointestinal standarization group. *Journal of Comparative Pathology*.

[8] Jenkins D., Balsitis M., Gallivan S. (1997). Guidelines for the initial biopsy diagnosis of suspected chronic idiopathic inflammatory bowel disease. The British Society of Gastroenterology Initiative. *Journal of Clinical Pathology*.

[9] Hall E. J., Lhermette P., Sobel D. (2008). Flexible endoscopy: basic technique. *Manual of Canine and Feline Endoscopy and Endosurgery*.

[10] Rychlik A. (2010). Przydatność wybranych wskaźników klinicznych, histopatologicznych i laboratoryjnych w ocenie aktywności nieswoistych zapaleń jelit u psów. *Medycyna Weterynaryjna*.

[11] Evans S. E., Bonczynski J. J., Broussard J. D., Han E., Baer K. E. (2006). Comparison of endoscopic and full-thickness biopsy specimens for diagnosis of inflammatory bowel disease and alimentary tract lymphoma in cats. *Journal of the American Veterinary Medical Association*.

[12] Gieger T. (2011). Alimentary lymphoma in cats and dogs. *Veterinary Clinics of North America: Small Animal Practice*.

[13] Simpson K. W., Jergens A. E. (2011). Pitfalls and progress in the diagnosis and management of canine inflammatory bowel disease. *Veterinary Clinics of North America: Small Animal Practice*.

[14] Jergens A. E., Moore F. M., Haynes J. S., Miles K. G. (1992). Idiopathic inflammatory bowel disease in dogs and cats: 84 cases (1987–1990). *Journal of the American Veterinary Medical Association*.

[15] German A. J., Helps C. R., Hall E. J., Day M. J. (2000). Cytokine mRNA expression in mucosal biopsies from German shepherd dogs with small intestinal enteropathies. *Digestive Diseases and Sciences*.

[16] Jergens A. E., Schreiner C. A., Frank D. E. (2003). A scoring index for disease activity in canine inflammatory bowel disease. *Journal of Veterinary Internal Medicine*.

[17] Washabau R. J., Day M. J., Willard M. D. (2010). Endoscopic, biopsy, and histopathologic guidelines for the evaluation of gastrointestinal inflammation in companion animals. *Journal of Veterinary Internal Medicine*.

[18] Willard M. D., Moore G. E., Denton B. D. (2010). Effect of tissue processing on assessment of endoscopic intestinal biopsies in dogs and cats. *Journal of Veterinary Internal Medicine*.

[19] McCann T. M., Ridyard A. E., Else R. W., Simpson J. W. (2007). Evaluation of disease activity markers in dogs with idiopathic inflammatory bowel disease. *Journal of Small Animal Practice*.

[20] Rychlik A., Nieradka R., Kander M., Nowicki M., Wdowiak M., Kołodziejska-Sawerska A. (2012). A correlation between the Canine Inflammatory Bowel Disease Activity Index score and the histopathological evaluation of the small intestinal mucosa in canine inflammatory bowel disease. *Polish Journal of Veterinary Sciences*.

[21] Rychlik A., Nieradka R., Kander M., Nowicki M., Wdowiak M., Kołodziejska-Sawerska A. (2013). The effectiveness of natural and synthetic immunomodulators in the treatment of inflammatory bowel disease in dogs. *Acta Veterinaria Hungarica*.

[22] Craven M., Mansfield C. S., Simpson K. W. (2011). Granulomatous colitis of boxer dogs. *Veterinary Clinics of North America: Small Animal Practice*.

[23] Maunder C. L., Reynolds Z. F., Peacock L., Hall E. J., Day M. J., Cogan T. A. (2016). Campylobacter species and neutrophilic inflammatory bowel disease in cats. *Journal of Veterinary Internal Medicine*.

[24] Willard M. D., Jergens A. E., Duncan R. B. (2002). Interobserver variation among histopathologic evaluations of intestinal tissues from dogs and cats. *Journal of the American Veterinary Medical Association*.

[25] Procoli F., Mõtsküla P. F., Keyte S. V., Priestnall S., Allenspach K. (2013). Comparison of Histopathologic findings in duodenal and ileal endoscopic biopsies in dogs with chronic small intestinal enteropathies. *Journal of Veterinary Internal Medicine*.

[26] Allenspach K. A., Mochel J. P., Du Y. (2019). Correlating gastrointestinal histopathologic changes to clinical disease activity in dogs with idiopathic inflammatory bowel disease. *Veterinary Pathology*.

[27] Allenspach K., Bergman P. J., Sauter S., Gröne A., Doherr M. G., Gaschen F. (2006). P-glycoprotein expression in lamina propria lymphocytes of duodenal biopsy samples in dogs with chronic idiopathic enteropathies. *Journal of Comparative Pathology*.

[28] Jacobs G., Collins-Kelly L., Lappin M., Tyler D. (1990). Lymphocytic-plasmacytic enteritis in 24 dogs. *Journal of Veterinary Internal Medicine*.

[29] Dennis J. S., Kruger J. M., Mullaney T. P. (1993). Lymphocytic/plasmacytic colitis in cats: 14 cases (1985–1990). *Journal of the American Veterinary Medical Association*.

[30] Baez J. L., Hendrick M. J., Walker L. M., Washabau R. J. (1999). Radiographic, ultrasonographic, and endoscopic findings in cats with inflammatory bowel disease of the stomach and small intestine: 33 cases (1990–1997). *Journal of the American Veterinary Medical Association*.

[31] Waly N. E., Stokes C. R., Gruffydd-Jones T. J., Day M. J. (2004). Immune cell populations in the duodenal mucosa of cats with inflammatory bowel disease. *Journal of Veterinary Internal Medicine*.

[32] Craven M., Simpson J. W., Ridyard A. E., Chandler M. L. (2004). Canine inflammatory bowel disease: retrospective analysis of diagnosis and outcome in 80 cases (1995–2002). *Journal of Small Animal Practice*.

[33] Tanaka H., Nakayama M., Takase K. (2003). Histiocytic ulcerative colitis in a French bulldog. *Journal of Veterinary Medical Science*.

[34] Simpson K. W., Dogan B., Rishniw M. (2006). Adherent and invasive *Escherichia coli* is associated with granulomatous colitis in boxer dogs. *Infection and Immunity*.

[35] Craven M., Dogan B., Schukken A. (2010). Antimicrobial resistance impacts clinical outcome of granulomatous colitis in boxer dogs. *Journal of Veterinary Internal Medicine*.

[36] Allenspach K., Culverwell C., Chan D. (2016). Long-term outcome in dogs with chronic enteropathies: 203 cases. *Veterinary Record*.

[37] Xavier R. J., Podolsky D. K. (2007). Unravelling the pathogenesis of inflammatory bowel disease. *Nature*.

[38] Chichkowski M., Hale L. P. (2008). Bacterial-mucosal interactions in inflammatory bowel disease—an alliance gone bad. *American Journal of Physiology-Gastrointestinal and Liver Physiology*.

[39] Kołodziejska-Sawerska A., Rychlik A., Depta A., Wdowiak M., Nowicki M., Kander M. (2013). Cytokines in canine inflammatory bowel disease. *Polish Journal of Veterinary Sciences*.

[40] Nicpoń J., Kubiak K., Sapikowski G., Dzimira S. (1999). Endoskopowa ocena wpływu wybranych niesterydowych leków przeciwzapalnych na śluzówkę żołądka u psów. *Medycyna weterynaryjna*.

[41] Kubiak K., Nicpoń J., Jankowski M., Nicpoń J., Spużak J. (2004). Wrzody żołądka u psów. *Medycyna weterynaryjna*.

[42] Curtsinger D. K., Carpenter J. L., Turner J. L. (1993). Gastritis caused by *Aonchotheca putorii* in a domestic cat. *Journal of the American Veterinary Medical Association*.

[43] Valentine B. A., McCarthy T. C. (2005). Endoscopic biopsy handling and histopathology. *Veterinary Endoscopy for the Small Animal Practitioner*.

[44] Bridgeford E. C., Marini R. P., Feng Y., Parry N. M., Rickman B., Fox J. G. (2008). Gastric Helicobacter species as a cause of feline gastric lymphoma: a viable hypothesis. *Veterinary Immunology and Immunopathology*.

[45] Meresse B., Ripoche J., Heyman M., Cerf-Bensussan N. (2009). Celiac disease: from oral tolerance to intestinal inflammation, autoimmunity and lymphomagenesis. *Mucosal Immunology*.

[46] Thalheim L., Williams L. E., Borst L. B., Fogle J. E., Suter S. E. (2013). Lymphoma Immunophenotype of dogs determined by immunohistochemistry, flow cytometry, and polymerase chain reaction for antigen receptor rearrangements. *Journal of Veterinary Internal Medicine*.

[47] Zandvliet M. (2016). Canine lymphoma: a review. *Veterinary Quarterly*.

[48] Kleinschmidt S., Harder J., Nolte I., Marsilio S., Hewicker-Trautwein M. (2010). Chronic inflammatory and non-inflammatory diseases of the gastrointestinal tract in cats: diagnostic advantages of full-thickness intestinal and extraintestinal biopsies. *Journal of Feline Medicine and Surgery*.

[49] Waly N. E., Gruffydd-Jones T. J., Stokes C. R., Day M. J. (2005). Immunohistochemical diagnosis of alimentary lymphomas and severe intestinal inflammation in cats. *Journal of Comparative Pathology*.

[50] Couto C. G., Rutgers H. C., Sherding R. G., Rojko J. (1989). Gastrointestinal lymphoma in 20 dogs. *Journal of Veterinary Internal Medicine*.

[51] Jergens A. E., Willard M. D., Day M. J., Tams T. R., Rawlings C. A. (2011). Endoscopic biopsy specimen collection and histopathologic considerations. *Small Animal Endoscopy*.

[52] Parachini-Winter C., Carioto L. M., Gara-Boivin C. (2019). Retrospective evaluation of anemia and erythrocyte morphological anomalies in dogs with lymphoma or inflammatory bowel disease. *Journal of the American Veterinary Medical Association*.

[53] MacDonald J. M., Mullen H. S., Moroff S. D. (1993). Adenomatous polyps of the duodenum in cats: 18 cases (1985–1990). *Journal of the American Veterinary Medical Association*.

[54] Slawienski M. J., Mauldin G. E., Mauldin G. N., Patnaik A. K. (1997). Malignant colonic neoplasia in cats: 46 cases (1990–1996). *Journal of the American Veterinary Medical Association*.

[55] Maas C. P. H. J., Ter Haar G., Van der Gaag I., Kirpensteijn J. (2007). Reclassification of small intestinal and cecal smooth muscle tumors in 72 dogs: clinical, histologic, and immunohistochemical evaluation. *Veterinary Surgery*.

[56] Willard M. D. (2012). Alimentary neoplasia in geriatric dogs and cats. *Veterinary Clinics of North America: Small Animal Practice*.

[57] Birchard S. J., Couto C. G., Johnson S. (1986). Nonlymphoid intestinal neoplasia in 32 dogs and 14 cats. *Journal of the American Animal Hospital Association*.

[58] Bray J., Dobson J. M., Lascelles D. (2011). Tumors of the colon and rectum. *Manual of Canine and Feline Oncology*.

[59] Mccaw D., Pratt M., Walshaw R. (1980). Squamous-cell carcinoma of the esophagus in a dog. *Journal of the American Animal Hospital Association*.

[60] Farese J. P., Bacon N. J., Ehrhart N. P., Bush J., Ehrhart E. J., Withrow S. J. (2008). Oesophageal leiomyosarcoma in dogs: surgical management and clinical outcome of four cases. *Veterinary and Comparative Oncology*.

[61] Knottenbelt C. M., Simpson J. W., Tasker S. (2000). Preliminary clinical observations on the use of piroxicam in the management of rectal tubulopapillary polyps. *Journal of Small Animal Practice*.

[62] Carpenter S., Petersen B. T., Chuttani R. (2007). Polypectomy devices. *Gastrointestinal Endoscopy*.

[63] Komeda Y., Kashida H., Sakurai T. (2017). Removal of diminutive colorectal polyps: a prospective randomized clinical trial between cold snare polypectomy and hot forceps biopsy. *World Journal of Gastroenterology*.

[64] Woods A., Sanowski R. A., Wadas D. D., Manne R. K., Friess S. W. (1989). Eradication of diminutive polyps: a prospective evaluation of bipolar coagulation versus conventional biopsy removal. *Gastrointestinal Endoscopy*.

[65] Peluso F., Goldner F. (1991). Follow-up of hot biopsy forceps treatment of diminutive colonic polyps. *Gastrointestinal Endoscopy*.

[66] Tappero G., Gaia E., De Giuli P., Martini S., Gubetta L., Emanuelli G. (1992). Cold snare excision of small colorectal polyps. *Gastrointestinal Endoscopy*.

[67] McAfee J. H., Katon R. M. (1994). Tiny snares prove safe and effective for removal of diminutive colorectal polyps. *Gastrointestinal Endoscopy*.

[68] Leib M. S., Saunders G. K., Moon M. L. (1993). Endoscopic diagnosis of chronic hypertrophic pyloric gastropathy in dogs. *Journal of Veterinary Internal Medicine*.

[69] Van Kruiningen H. J., Lees G. E., Hayden D. W., Meuten D. J., Rogers W. A. (1984). Lipogranulomatous lymphangitis in canine intestinal lymphangiectasia. *Veterinary Pathology*.

[70] Kull P. A., Hess R. S., Craig L. E., Saunders H. M., Washabau R. J. (2001). Clinical, clinicopathologic, radiographic, and ultrasonographic characteristics of intestinal lymphangiectasia in dogs: 17 cases (1996–1998). *Journal of the American Veterinary Medical Association*.

[71] Fan T. M., Simpson K. W., Polack E., Dykes N., Harvey J. (1999). Intestinal haemorrhage associated with colonic vascular ectasia (angiodysplasia) in a dog. *Journal of Small Animal Practice*.

[72] Daugherty M. A., Leib M. S., Lanz O. I., Duncan R. B. (2006). Diagnosis and surgical management of vascular ectasia in a dog. *Journal of the American Veterinary Medical Association*.

